# A self-regulatory cell-wall-sensing module at cell edges controls plant growth

**DOI:** 10.1038/s41477-024-01629-8

**Published:** 2024-03-07

**Authors:** Liam Elliott, Monika Kalde, Ann-Kathrin Schürholz, Xinyu Zhang, Sebastian Wolf, Ian Moore, Charlotte Kirchhelle

**Affiliations:** 1 Department of Plant Sciences, University of Oxford, Oxford, UK; 2 Laboratoire Reproduction et Développement des Plantes, Université Lyon 1, ENS de Lyon, CNRS, INRAE, Lyon, France; 3 Centre for Organismal Studies, University of Heidelberg, Heidelberg, Germany; 4 Center for Plant Molecular Biology, University of Tübingen, Tübingen, Germany

**Keywords:** Plant morphogenesis, Cell wall, Plant polarity, Protein trafficking in plants, Plant signalling

## Abstract

Morphogenesis of multicellular organs requires coordination of cellular growth. In plants, cell growth is determined by turgor pressure and the mechanical properties of the cell wall, which also glues cells together. Because plants have to integrate tissue-scale mechanical stresses arising through growth in a fixed tissue topology, they need to monitor cell wall mechanical status and adapt growth accordingly. Molecular factors have been identified, but whether cell geometry contributes to wall sensing is unknown. Here we propose that plant cell edges act as cell-wall-sensing domains during growth. We describe two Receptor-Like Proteins, RLP4 and RLP4-L1, which occupy a unique polarity domain at cell edges established through a targeted secretory transport pathway. We show that RLP4s associate with the cell wall at edges via their extracellular domain, respond to changes in cell wall mechanics and contribute to directional growth control in *Arabidopsis*.

## Abstract (web summary)

How do multicellular organisms integrate cell- and tissue-scale mechanical information to coordinate growth? Elliott et al. show that plant cells establish a self-regulating cell-wall-sensing module at their one-dimensional cell edges to control three-dimensional growth.

## Main

To develop defined organ shapes, adjacent cells need to coordinate their 3D growth. This can occur through tissue-scale organizing cues (morphogen gradients or stress fields), but at the local scale, heterogeneities in cellular growth can cause mechanical conflicts. In animal systems, such local conflicts can be relaxed through changes in tissue topology. In plants, cells are surrounded by a shared cell wall and cannot move relative to each other. Within the confines of this fixed tissue topology, mechanical conflicts have to be otherwise resolved.

Plant cell growth is driven by non-directional turgor pressure, which is translated into directional growth through construction and modification of a pecto-cellulosic cell wall with heterogeneous biochemical and mechanical properties^1,2^. Plants control growth direction primarily through oriented deposition of cellulose microfibrils of high tensile strength, which constrain growth parallel to their net orientation^3^ and are locally reinforced through interactions with hemicelluloses^4^. Pectins influence cell wall porosity but can also contribute to differential extensibility of the cell wall^5–7^. Despite their distinct structures and mechanical properties, the loss of specific cell wall components can be compensated by others. For example, pectins assume a more prominent load-bearing role in plant cell walls lacking the hemicellulose xyloglucan^8^. This implies that plant cells can perceive changes in their cell wall status and adapt their cell wall biogenesis accordingly. Several cell surface receptor families, including Wall-Associated Kinases (WAKs), *Catharanthus roseus* Receptor-Like Kinase 1-Likes (CrRLK1Ls) and Receptor-Like Proteins (RLPs), have been linked to cell wall sensing^9–14^.

Some of these receptors can directly interact with cell wall carbohydrates^6,15,16^, while in other cases, association with proteinaceous binding partners is required for downstream signalling events^17–20^. Despite the identification of such ligands, the role of these cell-wall-sensing systems in the continuous assembly and modification of the cell wall required during growth is not well understood. One reason for this may be a lack of appreciation of the spatial context (that is, the 3D geometry of the cell) in which such signals are perceived and translated into cell wall biogenesis.

Here we describe two *Arabidopsis thaliana* RLPs, RLP4 and RLP4-L1, that occupy a unique subcellular domain in the plasma membrane (PM) of growing cells: the geometric edges (where two faces of a polyhedral cell meet in a 1D line). We show that at the cell surface, RLP4s associate with the cell wall and respond to mechanical stimuli. We also show that surface-localized RLP4s contribute to directional growth control in *Arabidopsis* lateral roots through organizing edge-directed intracellular transport. On the basis of these data, we propose a mechanistic model for the translation of cell wall mechanical feedback into 3D growth through cell edges.

## Results

### Two RLPs localize to plant cell edges

**Fig. 1:**
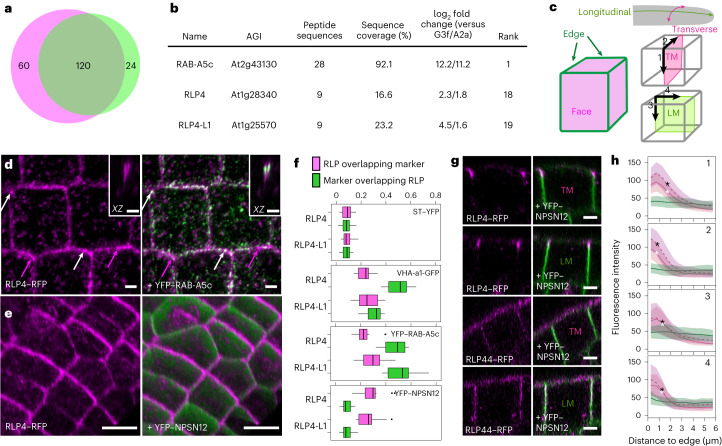
RLP4s are edge-polarized at the PM. **a**, Proteins enriched in the YFP–RAB-A5c interactome compared with YFP–RAB-A2a (magenta) and YFP–RAB-G3f (green). **b**, Ranking for RLP4s from the comparative proteomics approach. **c**, Schematic depiction of cell edges and midplane sections in lateral root epidermal meristem cells. TM, transverse midplane; LM, longitudinal midplane. **d**, Confocal laser scanning microscopy (CLSM) maximum-intensity projection of lateral root epidermal meristem cells co-expressing *pRLP4::RLP4–RFP* and YFP–RAB-A5c. The insets show *XZ* orthogonal sections at a cell edge. The experiments were conducted at least five times independently; representative images are shown. **e**, MorphographX snapshot of a lateral root meristem co-expressing *pUBQ10::RLP4–RFP* and YFP–NPSN12. **f**, Manders’s colocalization coefficients showing the fraction of RLP4s–RFP colocalizing with different membrane markers. CLSM stacks from three or four lateral root meristems were subdivided into non-overlapping substacks of 25 µm × 25 µm (‘regions’). *N* = 13 regions from four lateral roots (RLP4–RFP/VHA-a1–GFP) or 14 regions from three or four roots (all other combinations). See Methods for an explanation of the box plots. **g**, CLSM *XZ*/*YZ* projections representing TM and LM midplane sections through epidermal meristematic lateral root cells co-expressing *pUBQ10*::*RLP4–RFP* or *pUBQ10*::*RLP44–RFP* and YFP–NPSN12. The experiments were conducted at least five times independently; representative images are shown. **h**, Quantification of fluorescence intensity of RLP4–RFP (red), RLP4-L1–RFP (magenta, dashed line) and RLP44–RFP (green) with increasing distance from the cell edge along the trajectories labelled 1–4 in **c**. The lines indicate average fluorescence intensity in midplane sections with increasing distance from the edge. *N* for RLP4–RFP along trajectories 1–4 are 108, 123, 82 and 151, respectively. *N* for RLP4-L1–RFP for trajectories 1–4 are 103, 121, 92 and 162, respectively. The shaded areas indicate ±1 s.d. The asterisks indicate the distance from the cell edge at which RLP4–RFP (white asterisks) or RLP4-L1–RFP (black asterisks) signal intensity became significantly lower than at the edge (one-way ANOVA and post-hoc Tukey test, *P* < 0.05). Scale bars, 1 µm (**a**), 5 µm (**g**) or 10 µm (**e**). Source data

**Extended Data Fig. 1:**
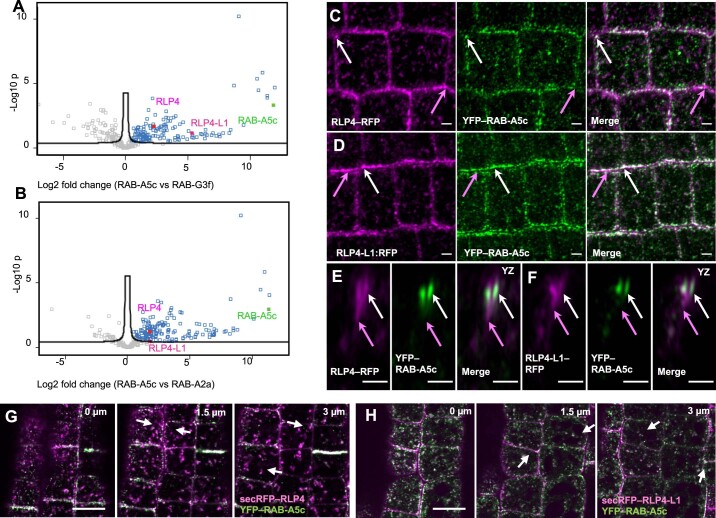
RLP4s contribute to directional growth control. (A,B) Volcano plots of the interactomes of YFP–RAB-A5c compared to YFP–RAB-G3f (A) and YFP–RAB-A2a (B). We conducted a two-sided volcano plot analysis with a S0 of 2 and FDR of 0.2 (non-adjusted for multiple comparisons), and proteins significantly enriched in the YFP–RAB-A5c interactome vs YFP–RAB-G3f or YFP–RAB-A2a in this analysis are colour-coded in blue. (C-F) CLSM maximum intensity (C,D) and YZ orthogonal projections (E,F) of lateral root epidermal meristem cells coexpressing *pRLP4s::RLP4s–RFP* (magenta) and *pRAB-A5c::YFP–RAB-A5c* (green). RLP4s–RFP colocalize with YFP–RAB-A5c at cell edge compartments (white arrows) and additionally label the peripheral cell edge (magenta arrows). Experiments were conducted at least 5 times independently, representative images are shown. (G,H) Sequential CLSM images of lateral root epidermal meristem cells co-expressing *pUBQ10::secRFP–RLP4s* (magenta) and *pRAB-A5c::YFP–RAB-A5c* (green). Experiments were conducted 3 times independently, representative images are shown. Scale bars 2 µm (C-F), 10 µm (G, H).

The plant-specific GTPase RAB-A5c mediates a transport pathway targeted to cell edges that is required for directional growth in *Arabidopsis* lateral roots^21^. We performed co-immunoprecipitation coupled with label-free semi-quantitative mass spectrometry against YFP–RAB-A5c^21^ to identify interactors of RAB-A5c. To separate generic Rab interactors from those specific to RAB-A5c, we identified proteins significantly enriched in the YFP–RAB-A5c interactome compared with the interactomes of two related Rab GTPases: the late endosome/tonoplast-localized YFP–RAB-G3f^22^ and the *trans*-Golgi network/early endosome (TGN/EE)-localized YFP–RAB-A2a^23^ (Supplementary Data 1, Fig. 1a and Extended Data Fig. 1a,b). In the top 20 candidates identified in this approach, we found two related proteins: RECEPTOR-LIKE PROTEIN4 (RLP4)^24^ and its closest relative in *Arabidopsis*, At1g25570, which we refer to as RECEPTOR-LIKE PROTEIN 4-LIKE1 (RLP4-L1) (Fig. 1b).

**Extended Data Fig. 2:**
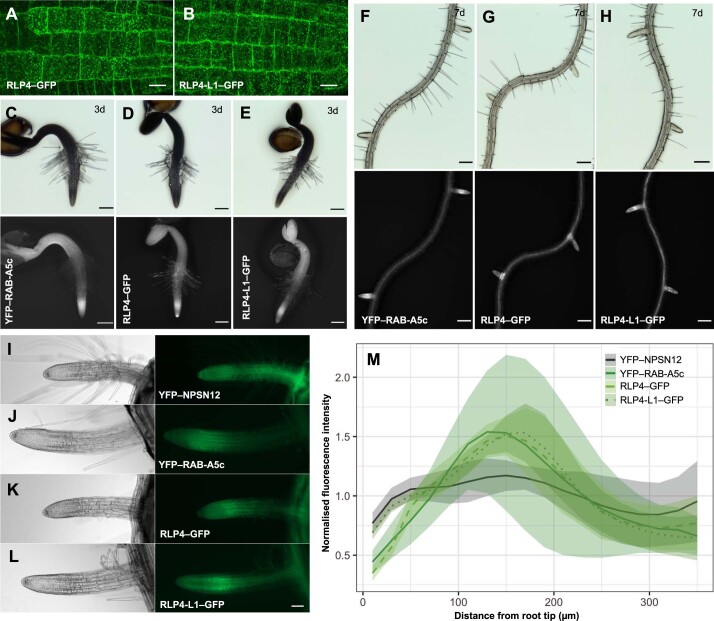
Expression pattern of RLP4 and RLP4-L1. (A,B) CLSM maximum intensity projections of lateral root epidermal meristematic cells expressing *pRLP4s::RLP4s–GFP*. Experiments were conducted at least 5 times independently, representative images are shown. (C-L) Brightfield and wide-field fluorescent images of 3 day old seedlings (C-E), 10 day old roots (F-H), or lateral roots from 10d old plants (I-L) expressing *pUBQ10::YFP–NPSN12*, *pRAB-A5c::YFP–RAB-A5c, pRLP4::RLP4–GFP*, or *pRLP4-L1::RLP4-L1–GFP*. Experiments were conducted 3 times independently, representative images are shown. (M) Quantification of fluorescence intensity with increasing distance from the root tip in lateral roots such as those in (I-L). Plots are mean intensity +/- 1 SD. n = 11 (YFP–NPSN12, YFP–RAB-A5c,RLP4-L1–GFP), 13 (RLP4–GFP). Scale bars 10 µm (A,B), 50 µm (I-L) or 100 µm (C-H).

Fluorescently tagged versions of RLP4 and RLP4-L1 (henceforth collectively referred to as RLP4s) under the control of their native promoters (*pRLP4s*::*RLP4s–GFP*) were functional (see the details below and in Fig. 4g and Extended Data Fig. 6e,f) and were highly expressed in growing tissues of the root and shoot (Extended Data Fig. 2a–l). Similarly to what has previously been described for *pRAB-A5c*::*YFP–RAB-A5c*^21^, expression in lateral roots was highest in epidermal meristem cells and was progressively reduced in differentiating cells (Extended Data Fig. 2m).

**Extended Data Fig. 3:**
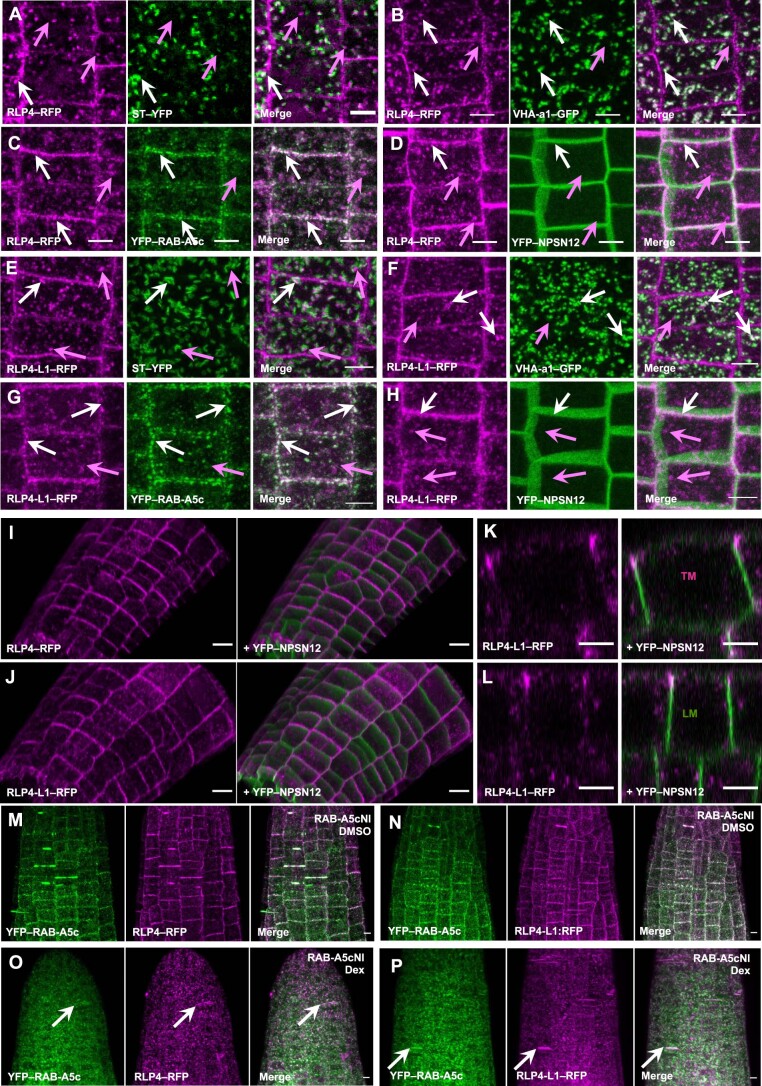
RLP4s are edge-restricted in Arabidopsis lateral root cells. (A-H) CSLM projections of lateral root epidermal meristem cells co-expressing pRLP4s::RLP4s–RFP or pUBQ10::RLP4s–RFP with various endomembrane markers. White arrows: colocalization of RLP4s–RFP with the respective endomembrane marker, magenta arrows: compartments uniquely labelled by RLP4s–RFP. Experiments were conducted 4 times independently, representative images are shown. (I,J) MorphoGraphX projections of lateral roots meristems co-expressing pUBQ10::RLP4s–RFP (magenta) and YFP–NPSN12 (green). Experiments were conducted at least 10 times independently, representative images are shown. (K,L) CLSM XZ/YZ projections representing transverse (TM; K) and longitudinal (LM; L) midplane sections through meristematic lateral root cells co-expressing pUBQ10::RLP4-L1–RFP (magenta) and YFP–NPSN12 (green). Experiments were conducted at least 10 times independently, representative images are shown. (M-P) CLSM maximum intensity projections of lateral root meristems co-expressing pUBQ10::RLP4s–RFP (magenta), YFP–RAB-A5c (green), and Dex-inducible dominant-negative AtRPS5a»DEX»RAB-A5c^N125I^ after 3d on DMSO (M,N) or 10 µM Dex (O,P). Arrows indicate cell plates. Experiments were conducted 2 times independently, representative images are shown. Scale bars 5 µm (A-H) or 10 µm (I-P).

At the cellular level, RLP4s expressed under either their native (*pRLP4s*::*RLP4s–RFP/ pRLP4s*::*RLP4s–GFP*) or the UBIQUITIN10 promoter (*pUBQ10*::*RLP4s–RFP*) labelled intracellular punctae as well as the cell periphery (Fig. 1d and Extended Data Fig. 1c–j). Quantitative colocalization analyses with a series of endomembrane compartment markers demonstrated that RLP4s–RFP localized in similar proportions to RAB-A5c edge compartments, the TGN/EE, and the PM, while labelling the Golgi to a lesser extent (Fig. 1e,f and Extended Data Fig. 3a–h). RLP4s–RFP were confined to a subdomain of the PM, which was apparent in our colocalization analyses (Manders’s colocalization coefficient, 0.09 ± 0.04 in both cases) as well as in orthogonal or 3D projections of confocal stacks, in which RLP4s–RFP were strikingly confined to cell edges (Fig. 1d,e,g,h and Extended Data Figs. 1e,f and 3i–l). This pattern differed significantly from that of RLP44–RFP, a related PM-localized RLP^25^ that does not label edge compartments (Fig. 1g,h). We have previously proposed that RAB-A5c mediates a secretory pathway from the TGN/EE to the PM at cell edges on the basis of the localization of nucleotide-free and constitutively active RAB-A5c variants to these compartments^21^. To test whether RLP4s–RFP are a cargo of RAB-A5c-mediated transport, we overexpressed dominant-negative RAB-A5c-N125I, which disrupts RAB-A5c function without inhibiting bulk secretory traffic^21^. In the presence of RAB-A5c-N125I, RLP4s–RFP were depleted from cell edge compartments and the PM (Extended Data Fig. 3m–p). We conclude that RLP4s reach the cell edge domain as cargos of RAB-A5c-mediated edge-directed transport, where they define a unique polarity domain.

### RLP4s interact with the cell wall at edges

**Fig. 2:**
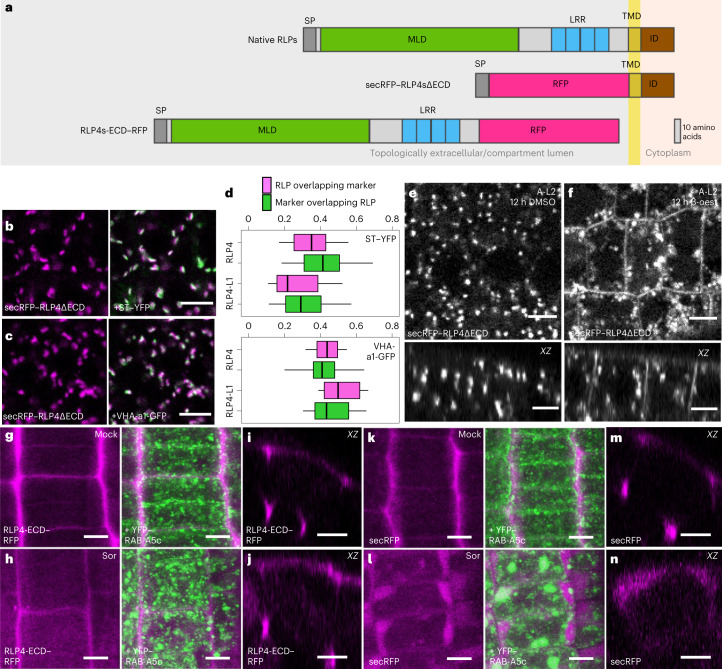
RLP4s associate with the cell wall. **a**, Schematic depiction of truncated RLP4 variants. MLD, malectin-like domain; LRR, leucine-rich repeat; TMD, transmembrane domain; ID, intracellular domain; SP, signal peptide. **b**,**c**, CLSM maximum-intensity projections of lateral root epidermal meristem cells co-expressing *pUBQ10*::*secRFP–RLP4ΔECD* with Golgi and TGN/EE markers. The experiments were conducted at least four times independently; representative images are shown. **d**, Manders’s colocalization coefficients showing the fraction of RLP4s–RFP colocalizing with the membrane markers shown in **b**,**c**. Three or more CLSM stacks of lateral root meristems per genotype were subdivided in 25 µm × 25 µm substacks (regions). *N* = 14 regions from three roots (secRFP–RLP4-L1ΔECD/VHA-a1–GFP), 15 regions from four roots (secRFP–RLP4ΔECD/VHA-a1–GFP) and 16 regions from four roots (all remaining combinations). See Methods for an explanation of the box plots. **e**,**f**, CLSM sections and *XZ* orthogonal projections of lateral root epidermal meristem cells co-expressing *pUBQ10*::*secRFP–RLP4ΔECD* and β-oestradiol-inducible A-L2 after 12 h of treatment with DMSO or 10 µM β-oestradiol (β-oest). The experiments were conducted three times independently; representative images are shown. **g**–**n**, CLSM maximum-intensity or *YZ* orthogonal projections of lateral root epidermal meristem cells co-expressing *pUBQ10*::*RLP4-ECD–RFP* or *pUBQ10*::*secRFP* and YFP–RAB-A5c after 30 minutes of incubation in H_2_O (mock) or 500 mM sorbitol (sor). The experiments were conducted three times independently; representative images are shown. Scale bars, 5 µm. Source data

RLP4s are predicted to contain a short intracellular domain, a transmembrane domain and an extracellular domain (ECD) containing leucine-rich repeats as well as a putatively carbohydrate-binding malectin-like domain also found in some CrRLK1Ls^24^ (Fig. 2a). The ECDs of other RLPs can interact with extracellular proteinaceous ligands or the cell wall^26^, whereas the intracellular domain is expected to interact with intracellular trafficking machinery.

**Extended Data Fig. 4:**
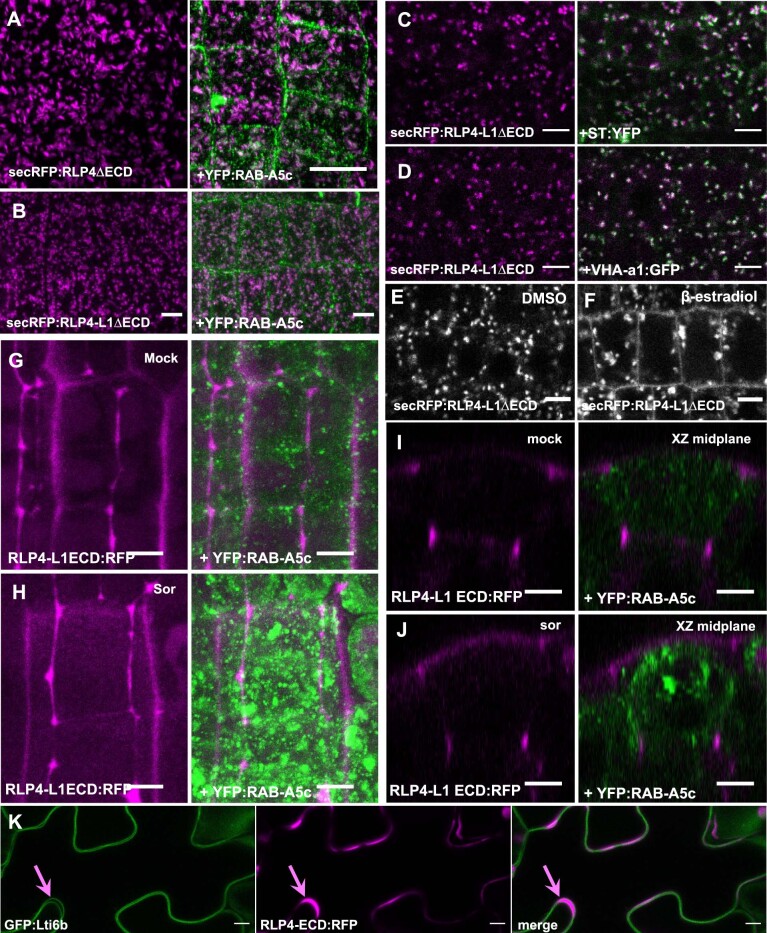
RLP4s associate with the cell wall. (A,B) CLSM maximum intensity projections of lateral root epidermal meristem cells co-expressing pUBQ10::secRFP–RLP4sΔECD and YFP–RAB-A5c. Experiments were conducted 3 times independently, representative images are shown. (C,D) CLSM maximum intensity projections of lateral root epidermal meristem cells co-expressing *pUBQ10::secRFP–RLP4-L1ΔECD* with Golgi and TGN/EE markers. Experiments were conducted 3 times independently, representative images are shown. (E,F) CLSM of lateral roots co-expressing *pUBQ10::secRFP–RLP4-L1ΔECD* and β-estradiol-inducible A-L2 after 12 h treatment with DMSO or 10 µM β-estradiol. Experiments were conducted 3 times independently, representative images are shown. (G-J) CLSM maximum intensity or YZ orthogonal projections of lateral roots co-expressing *pUBQ10::RLP4-L1-ECD–RFP* and YFP–RAB-A5c after 30 minutes incubation in H_2_O or 500 mM sorbitol. Experiments were conducted 3 times independently, representative images are shown. (K) CLSM single optical section of leaf epidermal pavement cells co-expressing *p35S::GFP–Lti6b* and *pUB10:RLP4-ECD–RFP*. Magenta arrow indicates RLP4-ECD–RFP localisation in the apoplast between plasma membranes of two cells labelled by GFP–Lti6b. Experiment was conducted 2 times independently, representative image is shown. Scale bars 5 µm.

To functionally characterize the ECD of RLP4s, we expressed RLP4s variants lacking their ECD fused to a secreted version of RFP (secRFP), targeting them to the secretory pathway (*pUBQ10*::*secRFP–RLP4sΔECD*; Fig. 2a). secRFP–RLP4sΔECD exclusively localized to intracellular compartments and did not label edge compartments or the PM (Fig. 2b,c and Extended Data Fig. 4a–d), while colocalization with Golgi and TGN/EE markers was significantly increased in comparison with full-length RLP4s–RFP (Fig. 2d versus Fig. 1f; *P* < 0.001, analysis of variance (ANOVA) and post-hoc Tukey test).

By contrast, full-length RLP4s with the equivalent amino-terminal tag (*pUBQ10*::*secRFP–RLP4s*) localized in the same pattern as carboxy-terminally tagged RLP4s (Extended Data Fig. 1g,h), suggesting that the N-terminal position of the tag did not interfere with protein transport. We hypothesized that secRFP–RLP4sΔECD may be secreted but undergo rapid endocytosis, preventing the accumulation of detectable levels at the PM. Consistent with this hypothesis, conditional overexpression of the clathrin uncoating factor AUXILIN-LIKE2 (A-L2), which causes specific inhibition of clathrin-mediated endocytosis^27^, resulted in partial relocalization of secRFP–RLP4sΔECD to the PM (Fig. 2e,f and Extended Data Fig. 4e,f).

We also expressed the RLP4s-ECD fused to RFP (*pUBQ10*::*RLP4s-ECD–RFP*). These truncations were secreted to the cell wall, with the strongest signal emanating from cell edges (Fig. 2g,i and Extended Data Fig. 4g,i). This pattern was also observed when secRFP was expressed on its own (*pUBQ10*::*secRFP*; Fig. 2k,m) and presumably reflects an inherent property of the cell wall rather than specific targeting of the protein to cell edges. In line with this interpretation, RLP4-ECD–RFP accumulated in the lobe regions of cotyledon pavement cells, where cell walls were thickest (Extended Data Fig. 4k). However, when we treated cells with 500 mM sorbitol for 30 min to plasmolyse them, secRFP flooded into the gap between the retracting protoplast and the cell wall (Fig. 2l,n), whereas RLP4s-ECD–RFP remained at the cell wall (Fig. 2h,j and Extended Data Fig. 4h,j). Taken together, our data show that the RLP4s-ECD can associate with the cell wall and are stabilized at the cell surface through this interaction.

### RLP4s respond to changes in cell wall mechanics

**Fig. 3:**
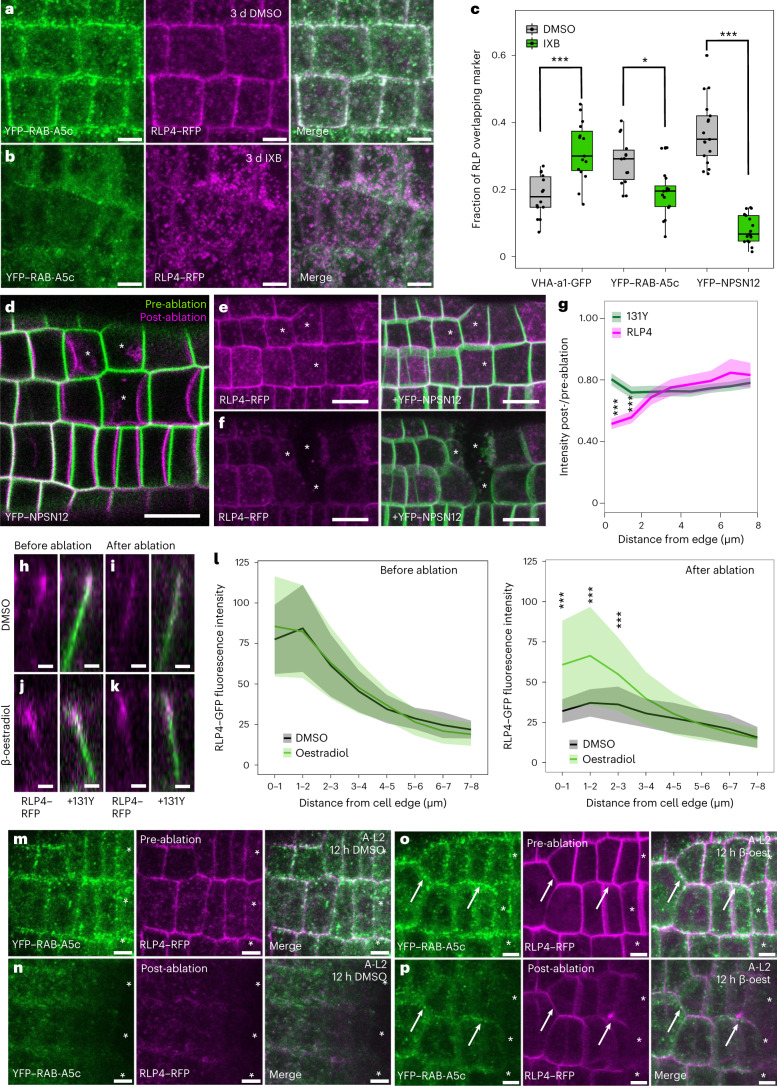
RLP4 responds to changes in cell wall mechanical status. **a**,**b**, CLSM maximum-intensity projections of lateral root epidermal meristems co-expressing *pUBQ10*::*RLP4–RFP* and *pRAB-A5c*::*YFP–RAB-A5c* after three days of treatment with 2.5 nM IXB or DMSO. **c**, Manders’s colocalization coefficients between RLP4s–RFP and various membrane markers with or without IXB as shown in **a**,**b**. *N* = 14 regions from two roots (VHA-a1–GFP DMSO and YFP–RAB-A5c DMSO), 15 regions from two roots (VHA-A1–GFP IXB), 16 regions from two roots (YFP–RAB-A5c IXB) or 17 regions from three roots (the remaining combinations). *P < 0.05; ***P < 0.001 (ANOVA with post-hoc Tukey test). See Methods for an explanation of the box plots. **d**–**f**, CLSM maximum-intensity projections of a lateral root epidermal meristem coexpressing RLP4–RFP and YFP–NPSN12 before and immediately after cell ablation (asterisks). **g**, Fluorescence intensity ratio of RLP4–RFP and YFP–NPSN12 after and before ablation on transverse midplane sections of epidermal meristem cells undergoing deformation after ablation like those shown in **d**–**f**. The lines indicate average values (*N* ≥ 62 edges from five roots), and the shaded areas show ±1 s.d. The asterisks indicate significant differences between RLP4–RFP and YFP–NPSN12 (one-way ANOVA and post-hoc Tukey test; ***P < 0.001). **h**–**k**, *XZ* projections showing the same anticlinal cell edges of lateral roots co-expressing *pUBQ10*::*RLP4–RFP*, YFP–NPSN12 and β-oestradiol-inducible A-L2 after 16 h of treatment with DMSO (**h**,**i**) or 10 µM β-oestradiol (**j**,**k**) before (**h**,**j**) and after (**i**,**k**) microneedle ablation. **l**, Fluorescence intensity of RLP4–RFP before and after ablation on midplane sections of epidermal meristem cells like those in **h**–**k**. The lines indicate average fluorescence intensity (*N* = 24 (DMSO) and *N* = 32 (β-oestradiol) edges from three roots, respectively), and the shaded areas show ±1 s.d. The asterisks indicate significant differences in RLP4–RFP intensity between DMSO and β-oestradiol treatments (one-way ANOVA and post-hoc Tukey test; ***P < 0.001). Before the ablation, there was no significant difference in RLP4–RFP intensity between treatments. **m**–**p**, CLSM maximum-intensity projections of lateral root epidermal meristem cells coexpressing RLP4–RFP and YFP–RAB-A5c in the absence (**m**,**n**) or presence (**o**,**p**) of inducible A-L2 before (**m**,**o**) and immediately after cell ablation (**n**,**p**; asterisks). Note that RLP4–RFP and YFP–RAB-A5c show increased retention at the cell edge in the presence of A-L2 (arrows). Scale bars, 10 µm (**d**–**f**), 5 µm (**a**,**b**,**m**–**p**) or 2 µm (**h**–**k**). Source data

**Extended Data Fig. 5:**
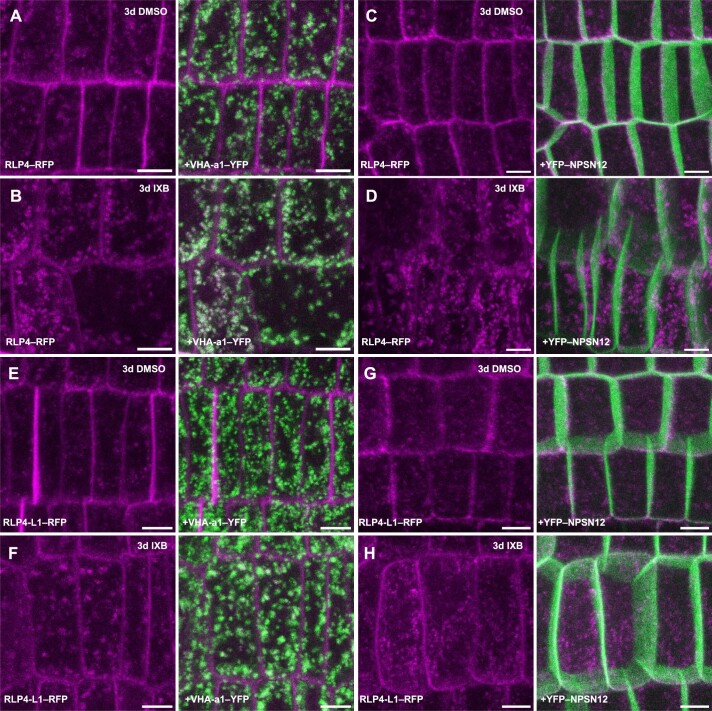
RLP4s localization is sensitive to cell wall perturbation. (A-H) CLSM maximum intensity projections of lateral root epidermal meristematic cells co-expressing *pUBQ10::RLP4s–RFP* and VHA-a1–GFP (A,B,E,F) or YFP–NPSN12 (C,D,G,H) after 3d on plates containing 2.5 nM IXB (B,D,F,H) or an equivalent quantity of DMSO (A),C,E,G). Experiments were conducted 2 times independently, representative images are shown. Scale bars 5 µm.

Considering their interaction with the cell wall, we hypothesized that RLP4s may act as cell wall sensors during growth. To test whether RLP4s are responsive to changes in cell wall mechanical status, we treated plants expressing RLP4s–RFP with isoxaben (IXB), an inhibitor of cellulose biosynthesis^28^. After three days of treatment with 2.5 nM IXB, RLP4–RFP was depleted from the PM and RAB-A5c-labelled compartments, whereas accumulation at the TGN/EE significantly increased (Fig. 3a–c). We observed a qualitatively similar albeit slightly weaker shift in localization from the PM towards intracellular compartments for RLP4-L1–RFP (Extended Data Fig. 5a–h).

We also noticed that YFP–RAB-A5c compartments were depleted from cell edges in IXB-treated roots (Fig. 3a,b), indicating that edge-directed transport itself was perturbed during IXB treatment. IXB acts through inhibiting the transport of cellulose synthase complexes to the PM^29^. This mode of action is believed to be due to specific interactions of IXB with the cellulose synthase subunits CESA3 and CESA6 (refs. ^28,30^), and trafficking of the PM-localized YFP–NPSN12 was not affected by IXB in our experiments (Extended Data Fig. 4c,d,g,h). However, IXB has been reported to perturb intracellular trafficking of the *endo*-1,4-β-*d*-glucanase KORRIGAN1 (ref. ^31^), and we could not exclude the possibility that the depletion of RLP4s from the cell surface was driven primarily by a perturbation of RLP4s transport to the cell surface rather than a direct response of RLP4s to cell wall status. To distinguish between the effects of IXB on RAB-A5c/RLP4s trafficking and those on surface retention of RLP4s, we employed an alternative strategy to perturb cell wall mechanical status that did not rely on long treatment periods.

We ablated small groups of cells with a microneedle to induce instantaneous local changes in cell geometry (Fig. 3d) and alterations of cell wall stress patterns surrounding the wound site^32^. We imaged lateral roots within five minutes before and after ablation, allowing us to follow the dynamics of RLP4s in response to mechanical perturbations with much higher temporal resolution. In these experiments, RLP4–RFP was significantly depleted from cell edges in the vicinity of ablations in comparison with the PM marker YFP–NPSN12 (Fig. 3e–g). To distinguish whether the loss of RLP4–RFP signal was due to loss of secretion or increased endocytosis of RLP4–RFP after ablation, we also conducted ablations in the presence of inducibly expressed A-L2 to inhibit endocytosis (Fig. 3h–p). After 16 h of induction, the RLP4–RFP pattern at cell edges was indistinguishable in A-L2-expressing and A-L2-non-expressing roots (Fig. 3h,j,l). However, after ablation, RLP4–RFP intensity at cell edges was significantly higher in A-L2-expressing roots (Fig. 3i,k,l), indicating that the observed reduction in RLP4–RFP under control conditions depended on endocytosis rather than secretion.

We conclude that RLP4s–RFP abundance at the surface changes in response to cell wall mechanical and/or biochemical status through enhanced endocytosis.

### RLP4s are required for RAB-A5c patterning and growth control

We also investigated localization patterns of YFP–RAB-A5c in ablation experiments and found that YFP–RAB-A5c was also lost from cell edges after ablations (Fig. 3m,n). When we conducted ablations in plants overexpressing A-L2, more YFP–RAB-A5c-labelled compartments persisted at cell edges in cells close to ablation sites (Fig. 3o,p), raising the question of whether RLP4s are directly involved in RAB-A5c recruitment to cell edges.

**Fig. 4:**
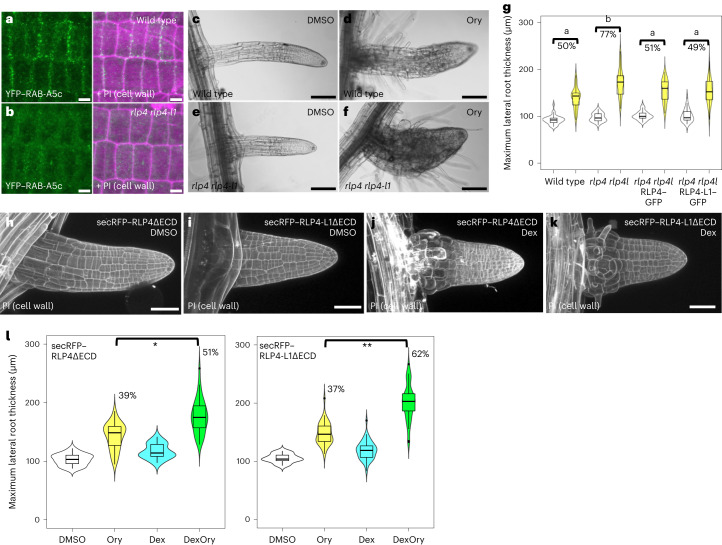
RLP4s contribute to directional growth control. **a**,**b**, CLSM maximum-intensity projections of lateral root epidermal meristem cells expressing YFP–RAB-A5c in the wild-type (**a**) or *rlp4* *rlp4-l1* (**b**) background. The cell wall was stained with propidium iodide (PI). Representative images from one of three independent experiments are shown. **c**–**f**, Lateral roots from wild-type and *rlp4* *rlp4-l1* plants grown for three days on DMSO or 250 nM oryzalin (Ory). **g**, Violin plots of the mean maximum diameter of lateral roots from plants grown for three days on DMSO or 250 nM oryzalin like those shown in **c**–**f** or S6F. *N* for DMSO and oryzalin for each genotype were 37 and 34 (wild type), 35 and 42 (*rlp4* *rlp4-l1*), 36 and 37 (*rlp4* *rlp4-l1 pRLP4*::*RLP4–GFP*) and 33 and 38 (*rlp4* *rlp4-l1 pRLP4-L1*::*RLP4-L1–GFP*). The difference in diameter (%) between DMSO and oryzalin treatments for each genotype is noted above the respective columns. Relative diameter increased significantly more in response to oryzalin treatment in *rlp4* *rlp4-l1* than in the wild type (*P* = 0.000007), which could be fully rescued by introducing *pRLP4*::*RLP4–GFP* (*P* = 1) or *pRLP4-L1*::*RLP4-L1–GFP* (*P* = 1) into the *rlp4* *rlp4-l1* background. The letters indicate significant differences in relative root diameter increase (*P* < 0.05; two-way ANOVA and post-hoc Tukey test). Representative results from one of three independent experiments are shown. See Methods for an explanation of the violin plots. **h**–**k**, CLSM maximum-intensity projections of lateral roots expressing *pRPS5a»Dex»secRFP–RLP4sΔECD* three days after transfer to DMSO or 10 µM Dex. The cell wall was stained with propodium iodide. **l**, Violin plots of the mean maximum diameter of lateral roots from seedlings expressing *pRPS5a»Dex»secRFP–RLP4sΔECD* grown on 250 nM oryzalin and/or 1 µM Dex or the equivalent quantity of DMSO for three days. *N* for DMSO, oryzalin, Dex and Dex oryzalin, respectively, were 30, 39, 18 and 34 (*RLP4ΔECD*); and 27, 37, 28 and 34 (*RLP4-L1ΔECD*). Relative diameter increases were significantly different for oryzalin treatments in the presence versus absence of Dex (*P = 0.038,**P = 0.012; two-way ANOVA and post-hoc Tukey test). Representative results from one of three independent experiments are shown. See Methods for an explanation of the violin plots. Scale bars, 5 µm (**a**,**b**), 50 µm (**h**–**k**) or 100 µm (**c**–**f**). Source data

**Extended Data Fig. 6:**
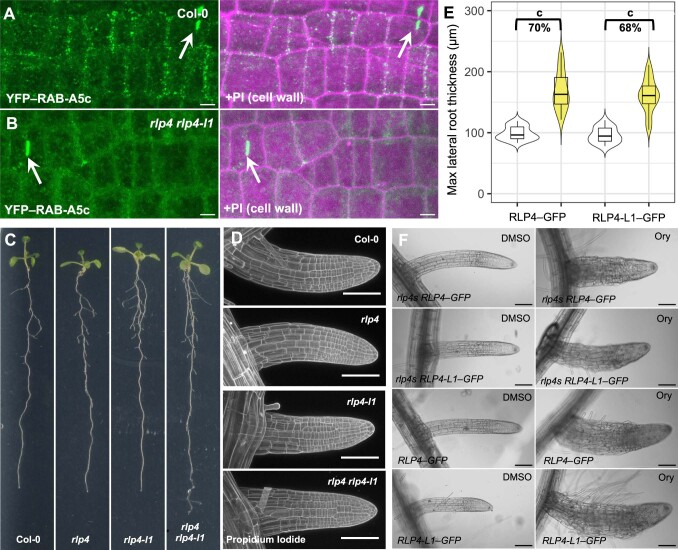
RLP4s are required for RAB-A5c localization. (A,B) CLSM maximum intensity projections of lateral root epidermal meristem cells expressing YFP–RAB-A5c in wild type (A) or *rlp4 rlp4-l1* (B) backgrounds. The cell wall was stained with propidium iodide. Cell plates are labelled with white arrows. Experiments were conducted 2 times independently, representative images are shown. (C) Photographs of 10d old Col-0, *rlp4*, *rlp4-l1*, and *rlp4 rlp4-l1* seedings. (D) CLSM maximum intensity projections of lateral roots from seedlings shown in (C). The cell wall was stained with propidium iodide. Experiments were conducted 2 times independently, representative images are shown. (E) Violin plots of the mean maximum diameter of lateral roots from plants after 3d treatment with DMSO or Oryzalin. N for plants treated with DMSO or Ory, respectively, was 32, 35 (*pRLP4::RLP4–GFP)* or 22, 37 (*pRLP4-L1::RLP4-L1–GFP)*. Difference in diameter (%) between DMSO (white) and Ory (yellow) treatments for each genotype noted above respective columns. Relative swelling in response to Ory is significantly increased compared to wild type plants (p = 0.005 and 0.028, respectively), but less so than in *rlp4 rlp4-l1* plants examined in the same experiment (Fig. 4G; p < 0.05, two-way ANOVA and post-hoc Tukey test). Experiment was conducted 3 times independently, and results from one representative experiment are shown. Violin plots are defined in the Methods section. (F) Representative images of lateral roots after 3d treatment with DMSO or Oryzalin from *rlp4 rlp4-l1 pRLP4::RLP4–GFP*, *rlp4 rlp4-l1 pRLP4-L1::RLP4-L1–GFP, pRLP4::RLP4–GFP, pRLP4-L1::RLP4-L1–GFP* plants as those in quantified in Fig. 4G, S6E. Experiments were conducted 3 times independently, representative images are shown. Scale bars 5 µm (A,B) or 100 µm (D,F).

To test this hypothesis, we used CRISPR–Cas9 to obtain transcriptional null *rlp4* *rlp4-l1* mutants. In *rlp4* *rlp4-l1* mutants, YFP–RAB-A5c was depleted from cell edges but not from cell plates (Fig. 4a,b and Extended Data Fig. 6a,b), indicating that RLP4s are required for RAB-A5c localization to cell edges during interphase. While the inhibition of RAB-A5c function causes severe growth defects^21^, *rlp4*, *rlp4-l1* and *rlp4* *rlp4-l1* were phenotypically indistinguishable from wild-type plants in standard growth conditions (Extended Data Fig. 6c,d). We have previously demonstrated that growth defects caused by the inhibition of RAB-A5c can be partially compensated through increased anisotropy of microtubule arrays, rendering RAB-A5c-N125I plants hypersensitive to the microtubule-depolymerizing drug oryzalin^33^. To test whether similar compensatory mechanisms may explain the lack of growth defects in *rlp4* *rlp4-l1* plants, we treated wild-type and *rlp4* *rlp4-l1* plants with oryzalin. We found significantly higher lateral root swelling in *rlp4* *rlp4-l1* than in wild-type lateral roots (Fig. 4c–g; 77% versus 50%, respectively), phenocopying oryzalin-treated RAB-A5c-N125I plants^33^. This phenotype was suppressed by the introduction of *pRLP4*::*RLP4–GFP* or *pRLP4-L1*::*RLP4-L1–GFP* into the *rlp4* *rlp4-l1* background, indicating that tagged versions of RLP4s were functional (Fig. 4g and Extended Data Fig. 6f). Interestingly, expression of the same protein variants in the wild-type background caused a significant increase in sensitivity to oryzalin compared with wild-type plants, although not to the same extent as *rlp4* *rlp4-l1* (Extended Data Fig. 6e,f). This suggests that plants are sensitive to the level of RLP4s, and overexpression as well as lack of RLP4s can lead to reduced growth robustness.

**Extended Data Fig. 7:**
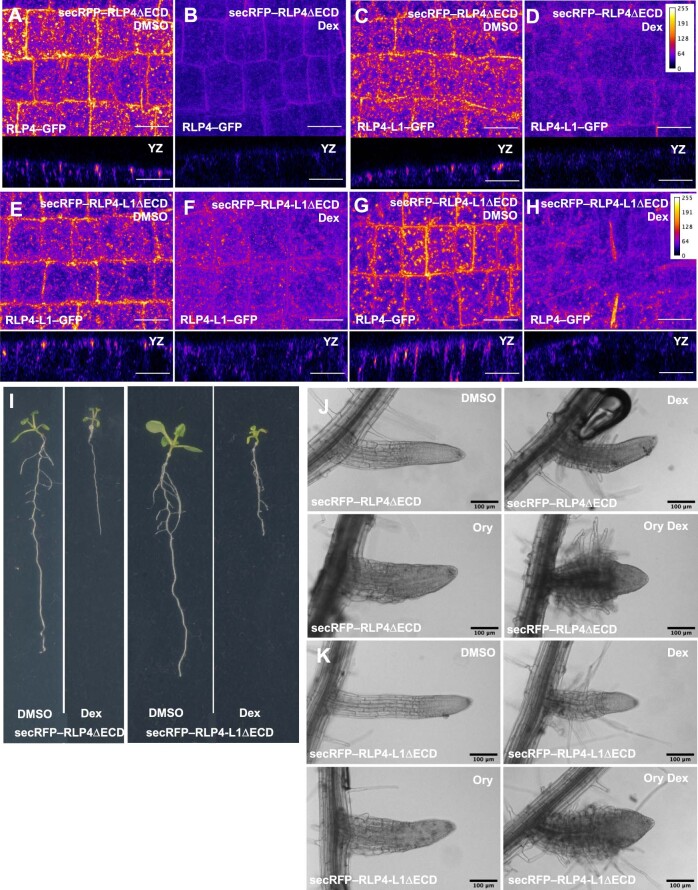
RLP4s are involved in edge-based growth control. (A-H) CSLM maximum intensity projections or YZ projections of lateral root epidermal meristem cells co-expressing *pRLP4s::RLP4s–GFP* and *pUBQ10::secRFP–RLP4sΔECD* 72 hours after transfer to DMSO (A,C,E,G) or 10 µM Dex (B,D,F,H). Images are displayed in the ImageJ “fire” LUT to emphasize differences in intensity. Experiments were conducted 3 times independently, representative images are shown. (I) Photographs of 10d old *pRPS5a»Dex»secRFP–RLP4sΔECD*, seedlings grown on 0.1% DMSO (left) or 10 µM Dex (right). (J,K) Brightfield images of lateral roots expressing *AtRPS5a»DEX»secRFP–RLP4sΔECD* 3 days after transfer to DMSO, 500 nM Dex (A,B) or 1 µM Dex (C), 250 nM Ory, 500 nM Dex + 250 nM Ory (A,B), or 1 µM Dex + 250 nM Ory (C). Experiments were conducted 3 times independently, representative images are shown. Scale bars 10 µm (A-H) or 100 µm (J,K).

We also aimed to conditionally disrupt RLP4s function and hypothesized that the overexpression of secRFP–RLP4sΔECD protein variants, which predominantly localized to the Golgi and TGN/EE (Fig. 2b–d and Extended Data Fig. 4a–d), may be used to disrupt the transport of wild-type RLP4s through competitive titration of intracellular trafficking machinery. We expressed these protein variants under the control of the dexamethasone (Dex)-inducible pOp/LhGR system^34^ (At*RPS5a»DEX»secRFP–RLP4sΔECD*) and found that secRFP–RLP4sΔECD overexpression strongly reduced the fluorescence of co-expressed *pRLP4s*::*RLP4s–GFP* at cell edges as well as intracellular compartments (Extended Data Fig. 7a–h). When induced from germination, secRFP–RLP4sΔECD caused growth defects reminiscent of those found in the roots and shoots of plants expressing RAB-A5c-N125I in 13/29 and 17/27 independent transgenic lines, respectively (Extended Data Fig. 7i).

When seven-day-old seedlings grown under non-inducing conditions were transferred to Dex for three days, lateral root morphology was strongly perturbed in secRFP–RLP4sΔECD plants (Fig. 4h–k). We have previously shown that RAB-A5c function is required for directional growth during interphase as well as cytokinesis, the latter of which is a function shared with other Rab-A GTPases^21^. By contrast, we observed no cytokinesis defects in At*RPS5a»DEX»secRFP–RLP4sΔECD* lines, indicating that RLP4s act specifically during interphase growth. Furthermore, At*RPS5a»DEX»secRFP–RLP4sΔECD* lines were hypersensitive to oryzalin (Fig. 4l and Extended Data Fig. 7j,k), phenocopying At*RPS5a»DEX»RAB-A5c*^N125I^ and *rlp4* *rlp4-l1*. We conclude that RLP4s control directional growth during interphase through tuning the delivery of RAB-A5c compartments to cell edges.

## Discussion

In this study, we identified and characterized two cell-wall-associated RLPs, RLP4 and RLP4-L1, which localize to cell edges, respond to changes in cell wall mechanics and are functionally linked to directional growth control. In growing tissues, 3D cellular growth is coordinated in different developmental zones but can vary substantially in neighbouring cells^33,35^. However, growth at shared 2D cell faces must be strictly synchronized to maintain tissue integrity. Even cell faces that are not shared (that is, at the outer organ surface) need to grow at appropriate rates to prevent cell bulging or rupture. 1D cell edges delimit cell faces in all directions, and requisite cell growth at any particular cell face can be considered as the product of integration of growth vectors along all edges delimiting the face. This implies that broader 2D and 3D growth patterns arise as a consequence of 1D growth control at cell edges.

**Fig. 5:**
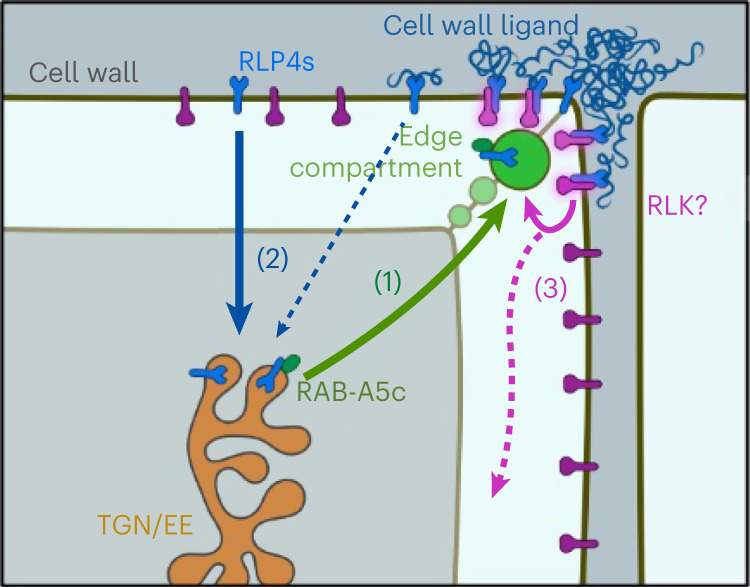
A hypothetical model for a self-regulating edge-based cell-wall-sensing module. (1) RLP4s are transported from the TGN/EE to the cell edge through RAB-A5c-mediated trafficking. (2) RLP4s are stabilized at the cell surface though interaction with a cell wall ligand, and the association of RLP4s with the cell wall is sensitive to changes in cell wall mechanical status. RLP4s that are not cell wall associated are endocytosed, thus providing a concentration-based system for cell wall sensing. (3) Surface-localized RLP4s associate with an as-yet-unidentified RLK to form a signalling module, among whose targets is RAB-A5c-mediated transport itself.

We and others have previously shown that cell edges are sites at which directional growth can be controlled^21,36^, but on the basis of the data presented here, we now propose cell edges simultaneously act as cell-wall-sensing domains through which cell wall mechanical status can be perceived and integrated into directional growth control (Fig. 5). We propose that (1) RAB-A5c mediates the delivery of RLP4s to the cell edge domain, where RLP4s associate with a cell wall ligand via their ECD; and (2) RLP4s abundance at the cell surface is constantly adapted through the removal of non-cell-wall-associated RLP4s through endocytosis, which allows rapid response to changes in cell wall status. RLP4s lack an intracellular kinase domain to initiate a downstream signalling cascade, and we currently do not know any interaction partners at the cell surface. However, other PM-localized RLPs interact with RLKs to form signalling modules that initiate intracellular signal cascades^25,37,38^. We therefore propose (3) a similar mode of action for RLP4s, which may act as a scaffold for an edge-based signalling hub whose activity can be controlled through RLP4s abundance at the edge. While we have not yet identified a direct target of such a module, our data show that RAB-A5c is among the downstream effectors of RLP4s, thus forming a positive feedback loop of edge-based growth control. This model can explain how cell wall mechanical status can be integrated into directional growth control through 1D cell edges.

We have developed this framework of edge-based growth control in plant tissues. However, there are many conceptual parallels to epidermal tissues in animals. In such tissues, tricellular junctions (anticlinal edges) have been implicated in responses to mechanical stimuli and also accumulate components of the JNK and Hippo growth signalling pathways^39^. This raises the intriguing possibility that growth control mechanisms in multicellular organisms of different lineages converge on 1D cell edges as regulatory domains.

## Methods

### Plant materials and growth

The *A. thaliana* ecotype Columbia (Col-0) was used throughout. The following transgenic lines used in this study have been described before: *pRAB-A5c*::*YFP–RAB-A5c*^21^, At*RPS5a»Dex»RAB-A5c*^N125I^ (ref. ^21^), *pUBQ10*::*YFP–NPSN12* (ref. ^22^), *pUBQ10*::*YFP–RAB-G3f*^22^, *pRAB-A2a*::*YFP–RAB-A2a*^23^, *pVHA-a1*::*VHA-a1–GFP*^40^, *p35S*::*ST–YFP*^41^ and *XVE»AL1/XVE»AL2* (ref. ^27^).

For the simultaneous targeting of *RLP4* and *RLP4-L1* via CRISPR–Cas9, two suitable sequences for the generation of guide RNAs were determined using the ChopChop webpage (https://chopchop.cbu.uib.no/) and incorporated into oligonucleotides that also contained an Eco31I recognition site at the 5′ end and a pHEE2E-TRI-specific^42^ sequence at the 3′ end. pHEE2E-TRI was used as a template to amplify the two targeting sequences together with the promoter and terminator regions. The amplified PCR product was gel-purified and ligated into Eco31I (BsaI)-digested pHEE2E-TRI. The assembled construct was mobilized in *Agrobacterium tumefaciens* strain GV3101 and used to transform Col-0 plants. T_1_ plants were selected on half-strength Murashige and Skoog (MS) medium containing 0.75% phytoagar and 15 μg ml^−1^ hygromycin. The plates were covered with sheets of paper for four to six days until positive T_1_ plants with an elongated hypocotyl could be distinguished and kept for another four days at full light. Around 40 T_1_ plants were transferred to soil and analysed for mutations using primers. We isolated a Cas9-free double mutant with single base insertions in both genes (position 264 from ATG for RLP4 and position 363 for RLP4-L1), leading to premature stop codons 14 and 11 exons downstream, respectively.

All plants were grown at 20 °C in a 16 h:8 h day:night cycle. Lateral roots were imaged 8–12 days after germination on upright half-strength MS medium (Sigma Aldrich) plates with 1% w/v sucrose and 0.8% Bacto agar (Appleton Woods) at pH 5.7. For conditional expression using either Dex or β-oestradiol, seedlings were grown for seven days from germination before transfer to half-strength MS medium containing either 10 µM Dex (Sigma Aldrich; diluted from a 10 mM stock in DMSO), 10 µM β-oestradiol (Sigma Aldrich; diluted from 10 mM a stock in DMSO) or an equivalent volume of DMSO solvent for the indicated period. Plasmolysis was performed by immersion of plants in 0.5 M sorbitol solution for 30 minutes. For pharmacological treatments with oryzalin or IXB, seedlings were grown for seven days from germination before transfer to half-strength MS medium containing either 2.5 nM IXB (Sigma Aldrich), 250 nM oryzalin (Sigma Aldrich; diluted from a 10 mM stock in DMSO) or an equivalent volume of DMSO solvent for the indicated period.

The introduction of novel transgenes into plants was performed using *Agrobacterium*-mediated floral dip transformation^43^.

### Molecular cloning

All genes were amplified by PCR using Phusion High-Fidelity DNA Polymerase (Thermo Fisher Scientific) from genomic DNA isolated from *A. thaliana* ecotype Col-0. *pUBQ10*::*RLP4/4-L1–RFP*, *pUBQ10*::*RLP44–RFP* and *pUQ10B*::*RLP4/4-L1-ECD–RFP* were all generated by cloning the relevant genomic DNA region into pDONR207 (Invitrogen/Thermo Fisher Scientific) using Gateway BP Clonase II Enzyme Mix (Thermo Fisher Scientific) and subsequently into *pUB–RFP–DEST* (*9*) using Gateway LR Clonase II Enzyme Mix (Thermo Fisher Scientific). For expression of RLP4–RFP and RLP4-L1–RFP from their native promoters, the UBQ10 promoter was removed from *pUB–RFP–DEST* through digestion with restriction endonucleases PspXI and PmeI (New England Biolabs), and the vector was subsequently re-ligated using Klenow polymerase (DNA Polymerase I, Large fragment; New England Biolabs) and T4 DNA ligase (Thermo Fisher Scientific) to generate *pX–DEST–RFP*. The promoter region, 5′ untranslated region and coding region of RLP4 and RLP4-L were then amplified by PCR as single cassettes and cloned into *pDONR207* and eventually *pX–DEST–RFP* as described above. To generate *pUBQ10*::*secRFP–RLP4s* and *pUBQ10*::*secRFP–RLP4sΔECD*, the relevant genomic DNA regions were overlapped with *secRFP*^44^ and the cassettes cloned into *pENTR/D-TOPO* using a pENTR/D-TOPO Cloning Kit (Thermo Fisher Scientific) and subsequently *pUB–DEST*^45^. For conditional expression of RLP4s and truncated variants using the pOp/LhGR system, transgenes were cloned into *pDONR207* using Gateway BP Clonase II Enzyme Mix (Thermo Fisher Scientific) and subsequently into *pOpIN2–RPS5a*^34^ using Gateway LR Clonase II Enzyme Mix (Thermo Fisher Scientific). All constructs were verified by Sanger sequencing (Source Bioscience) and restriction digests. For molecular cloning, *Escherichia coli* strains DH5α and DB3.1 were used. For *Agrobacterium*-mediated transformation of *Arabidopsis*, constructs were introduced into *Agrobacterium tumefaciens* strain GV3101::pMP90 by electroporation.

List of primers:

**Table:** 

Primer name	Sequence	Used to generate
RLP4_GW_F	GGGGACAAGTTTGTACAAAAAAGCAGGCTTCACCATGATGCTTCGATTTATCCTAGCTTCTCTTCTC	pUBQ10::RLP4–RFP pUBQ10::RLP4-ECD–RFP
RLP4_GW_R	GGGGACCACTTTGTACAAGAAAGCTGGGTCAGACAACAAGCTCGGTCCATTTTCCAC	pUBQ10::RLP4–RFP pUBQ10::RLP4-ECD–RFP pRLP4::RLP4–GFP
RLP4L1_GW_F	GGGGACAAGTTTGTACAAAAAAGCAGGCTTCACCATGCCCTTCTCTCCTTCCTTCTTC	pUBQ10::RLP4-L1–RFP pUBQ10::RLP4-L1-ECD–RFP
RLP4L1_GW_R	GGGGACCACTTTGTACAAGAAAGCTGGGTCTTGCGAATTCAGTGGAAGAGTGGGC	pUBQ10::RLP4-L1–RFP pUBQ10::RLP4-L1-ECD–RFP pRLP4L1::RLP4-L1–GFP
RLP4_ECD_GW_R	GGGGACCACTTTGTACAAGAAAGCTGGGTCCTTGGCTCCAGAAGAAAGGTGAGGC	pUBQ10::RLP4-ECD–RFP
RLP4L1_ECD_GW_R	GGGGACCACTTTGTACAAGAAAGCTGGGTCTTTACCCCCTTTGGATAAG	pUBQ10::RLP4-L1-ECD–RFP
RLP4_pro_GW_F	GGGGACAAGTTTGTACAAAAAAGCAGGCTTCACCAATTTAAAACACCTAAGGAGTGCACATACGGTCGAGCTAGAGAAGAGTAGAG	pRLP4::RLP4–GFP
RLP4L1_pro_GW_F	GGGGACAAGTTTGTACAAAAAAGCAGGCTTCACCCTAAACAAAACTACCACGAGCTTAAGACTGAATGGAGAGGATAAGGAGAGGTG	pRLP4L1::RLP4-L1–GFP
secRFP_GW_F	GGGGACAAGTTTGTACAAAAAAGCAGGCTTCACCATGAAGACTAATCTTTTTCTCTTTCTCATCTTTTCACTTCTC	pUBQ10::secRFP pUBQ10::secRFP–RLP4 pUBQ10::secRFP–RLP4-L1 pUBQ10::secRFP–RLP4ΔECD pUBQ10::secRFP–RLP4-L1ΔECD AtRPS5a»DEX»RLP4ΔECD AtRPS5a»DEX»RLP4-L1ΔECD
secRFP_GW_R_STOP	GGGGACCACTTTGTACAAGAAAGCTGGGTCTTAGGCGCCGGTGGAGTG	pUBQ10::secRFP
secRFP_R_LINKER	AGCTCCTCCAGCTCCTCCGGCGCCGGTGGAGTGGCG	pUBQ10::secRFP–RLP4 pUBQ10::secRFP–RLP4-L1 pUBQ10::secRFP–RLP4ΔECD pUBQ10::secRFP–RLP4-L1ΔECD AtRPS5a»DEX»RLP4ΔECD AtRPS5a»DEX»RLP4-L1ΔECD
RLP4_TMD_F_LINKER	GGAGGAGCTGGAGGAGCTATTGGCATTGCATTCGG	pUBQ10::secRFP–RLP4 pUBQ10::secRFP–RLP4ΔECD AtRPS5a»DEX»RLP4ΔECD
RLP4L1_TMD_F_LINKER	GGAGGAGCTGGAGGAGCTATAGCCATAGCCATATC	pUBQ10::secRFP–RLP4-L1 pUBQ10::secRFP–RLP4-L1ΔECD AtRPS5a»DEX»RLP4-L1ΔECD
RLP44_GW_F	GGGGACAAGTTTGTACAAAAAAGCAGGCTTCACCATGACAAGGAGTCACCGGTTAC	pUBQ10::RLP44–RFP
RLP44_GW_R	GGGGACCACTTTGTACAAGAAAGCTGGGTCGTAATCAGGCATAGATTGACTAATCTTACCTTC	pUBQ10::RLP44–RFP
RLP4_GW_R_STOP	GGGGACAAGTTTGTACAAAAAAGCAGGCTTCACCTCAAGACAACAAGCTCGGTC	pUBQ10::secRFP–RLP4 pUBQ10::secRFP–RLP4ΔECD AtRPS5a»DEX»RLP4ΔECD
RLP4L1_GW_R_STOP	GGGGACAAGTTTGTACAAAAAAGCAGGCTTCACCCTATTGCGAATTCAGTGGAAGAGTG	pUBQ10::secRFP–RLP4-L1 pUBQ10::secRFP–RLP4-L1ΔECD AtRPS5a»DEX»RLP4-L1ΔECD
RLP4_geno_CRISPR_F	GGATTAGTTGTGGAGCTAG	*rlp4* *rlp4-l1* plant lines
RLP4_geno_CRISPR_F	TTGACTACTCCAACCAGATT	*rlp4* *rlp4-l1* plant lines
RLP4L1_geno_CRISPR_F	AAACTGAATTCTTCCTCTGTT	*rlp4* *rlp4-l1* plant lines
RLP4L1_geno_CRISPR_R	ATCTCCAAGAGAAAACAAGAG	*rlp4* *rlp4-l1* plant lines

### Protein extraction and proteomics

Co-immunoprecipitation and mass spectrometry for the identification of interactors of YFP–RAB-A5c, YFP–RAB-A2a and YFP–RAB-G3f were performed as previously described^46^. In brief, the co-immunoprecipitation experiments were carried out by isolating total microsomes from *Arabidopsis* roots expressing YFP–RAB-A5c, YFP–RAB-A2a and YFP–RAB-G3f, or no transgene (Col-0). In-gel trypsin digest and mass spectrometry were performed by the Central Proteomic Facility, University of Oxford (www.proteomics.ox.ac.uk), and label-free quantification of the proteome was performed on three biological replicates using the SinQ pipeline^47^. We excluded all proteins that did not occur in all three replicates of YFP–RAB-A5c, replaced all remaining zero values in the matrix with the half-minimum value across all detected proteins and analysed the resulting 315 proteins for enrichment in RAB-A5c versus RAB-A2a and RAB-G3f proteomes using the Volcano plot function in the Perseus computational platform^48^, with an S0 of 2 and FDR of 0.2, which identified 120 proteins significantly enriched in the YFP–RAB-A5c interactome compared with both YFP–RAB-A2a and YFP–RAB-G3f. We ranked these according to four criteria: (1) abundance in the YFP–RAB-A5c interactome (descending order), (2) relative enrichment against the YFP–RAB-A2a interactome (descending order), (3) relative enrichment against the YFP–RAB-G3f interactome (descending order) and (4) abundance in the Col-0 negative control (ascending order). We then assigned a super rank according to the sum of individual ranks in ascending order (Supplementary Data 1).

### Microscopy and image analysis

Confocal microscopy was performed using a Zeiss 880 CLSM using a C-Apochromat ×40/1.20 W Corr M27 objective or a Zeiss 980 CLSM using a C-Apochromat ×40/1.20 W Corr M27 objective. GFP, YFP, RFP and PI were imaged as described before^23^. Image analysis and processing (orthogonal sectioning, maximum-intensity projections, image assembly and quantification) were performed using Fiji v. 2.14.0 (ref. ^49^). For the quantification of colocalization between RLP4s–RFP and various endomembrane markers, CLSM stacks of lateral roots were subdivided in 25 µm × 25 µm substacks of meristematic epidermal cells. These areas were chosen to allow the assessment of tissue-scale differences in localization pattern as well as root-to-root differences. Background signal was removed using a hysteresis filter^49^, using thresholds based on mean and minimum intensity minus 2 s.d. of ten randomly measured compartments for the respective CLSM channel, and Manders’s colocalization coefficients^50^ were determined using JACoP (Just Another Colocalisation Plugin) in Fiji v. 2.14.0 (ref. ^51^). Differences between different substacks from the same root were larger than differences between roots, and we pooled substacks from three or four lateral root stacks acquired during the same experiment. All experiments were conducted at least twice independently, and quantifications for one representative experiment are shown.

For the quantification of RLP4s–RFP at the PM, CLSM stacks of lateral roots co-expressing pUBQ10::YFP–NPSN12 and pUBQ10::RLP4s–RFP or pUBQ10::RLP44–RFP were collected at Nyquist resolution (voxel size, 99.5 nm × 99.5 nm × 550 nm). Midplane transverse and longitudinal sections of meristematic cells were generated in Fiji, and cellular outlines were manually traced using the PM marker YFP–NPSN12 as a reference. A plot profile with a width of seven pixels was generated, and RFP intensity was measured along the profile. The average signal intensity for ≥82 edges from meristematic epidermal cells of three or four lateral roots was calculated for 0.5-µm-wide intervals starting at the edge for longitudinal anticlinal, transverse anticlinal, longitudinal periclinal and transverse periclinal walls. The average intensity ± s.d. was plotted using the ggplot2 function in R Studio v. 4.1.2 (ref. ^52^). For the ablation experiments, 3D confocal stacks were acquired before and immediately after ablation. For quantitative analysis, only cell walls that were visibly deformed due to the ablation within a distance of six cells from the wound site were considered. RLP4–RFP and YFP–NPSN12 intensity along midplane sections of the same walls were quantified before and after ablation as described above, and the ratio post-/pre-ablation was calculated for each wall. The average ratio ± s.d. was plotted using the ggplot2 function in R Studio v. 4.1.2.

To quantify root thickness, we acquired bright-field images of lateral roots between 200 µm and 800 µm long and ensured that the mean root length was not significantly different across genotypes that were compared. The images were imported into Fiji v. 2.14.0, both sides of the root were traced manually along their longitudinal axis and *XY* Cartesian coordinates for each pixel on the outline trace were exported as .csv files and imported into RStudio v. 4.1.2 (https://www.rstudio.com/). For each pixel on one side, its closest neighbour on the other side was determined, and the Euclidian distance between pixels was calculated using the nn2 function in the RANN package (https://CRAN.R-project.org/package=RANN). The maximum diameter of each root was calculated as the average of the ten largest values excluding the tip-most 100 µm of each root to exclude the tapering tip. All experiments were conducted at least two times independently, and quantitative data from one representative experiment are shown.

### Statistical data analysis and reproducibility

Two-way ANOVA was performed in R using the aov function from the stats package^53^. Tukey’s test was performed in R using the TukeyHSD function from the stats package, and Student’s *t*-test was performed in R using the t.test function from the stats package. The box, ribbon and violin plots were generated in R using the ggplot2 function^52^. In the box plots, the median is displayed as a horizontal line, the lower and upper edges correspond to the 25th and 75th percentiles, and the lower and upper whiskers extend from the edges to the smallest or largest value no further than 1.5× the interquartile range from the edge. Data beyond the ends of the whiskers are plotted individually. The violin plots show the same information as the box plots, with the addition of the kernel probability density of the data at different values. The ribbon plots show the data mean ± s.d. (shaded areas).

All experiments were conducted at least twice and up to six times independently (see the details for specific experiments in the figure legends). For experiments involving confocal images of lateral roots, 3–8 lateral roots were imaged for each condition/genotype in each experimental repeat; for experiments involving bright-field images, 18–30 lateral roots were imaged in each experimental repeat. Data from one representative experiment are shown.

### Reporting summary

Further information on research design is available in the Nature Portfolio Reporting Summary linked to this article.

### Supplementary information

Reporting Summary

Supplementary Data 1 Ranked list of RAB-A5c interactor candidates from comparative proteomics.

### Source data

Source Data Fig. 1 Statistical source data.

Source Data Fig. 2 Statistical source data.

Source Data Fig. 3 Statistical source data.

Source Data Fig. 4 and Extended Data Fig. 6 Statistical source data.

Source Data Extended Data Fig. 2 Statistical source data.

## Data Availability

The data supporting the findings of this study are available within the Article and its Supplementary Information. The full proteomics dataset used in this study has been deposited at the PRIDE database under the title ‘Comparative proteomic identification of Rab GTPase interactors in Arabidopsis’, accession no. PXD044263. Source data are provided with this paper.
